# Non-canonical WNT6/WNT10A signal factor expression in EBV+ post-transplant smooth muscle tumors

**DOI:** 10.1186/s13569-018-0096-8

**Published:** 2018-06-04

**Authors:** Kristin Teiken, Mark Kuehnel, Jan Rehkaemper, Hans Kreipe, Florian Laenger, Kais Hussein, Danny Jonigk

**Affiliations:** 1 Institute of Pathology, Hannover Medical School (MHH), Carl-Neuberg-Str. 1, 30625 Hannover, Germany; 2 Institute of Pathology, University of Muenster, Domagkstraße 17, 48149 Muenster, Germany

**Keywords:** PTSMT, Post-transplant smooth muscle tumors, Angioleiomyomas, EBV, WNT

## Abstract

Post-transplant smooth muscle tumors (PTSMTs) are rare mesenchymal neoplasms which occur after solid organ or haematopoietic stem cell transplantation. PTSMT typically consist of Epstein–Barr-virus (EBV)+ smooth muscle-like cells and show an intermediate malignancy. Their main occurrences are visceral organs, especially the liver, but intracranial appearances are described and associated with a poor prognosis. EBV drives the growth of PTSMT; however, the underlying molecular mechanisms still remain unclear. Gene expression analysis of a set of morphologically similar tumors (leiomyomas, leiomyosarcomas, angioleiomyomas and endothelial haemangiomas) from patients without immunosuppression or EBV-association was performed. Our findings indicate that PTSMT’s growth is driven by two factors of the wingless-type protein family: WNT6 and WNT10A. We are first to report that in PTSMTs, a non-canonical activation of WNT, independent of beta-catenin, drives tumor cell proliferation via MTOR/AKT1, MYC and Cyclin D2.

**Electronic supplementary material:**

The online version of this article (10.1186/s13569-018-0096-8) contains supplementary material, which is available to authorized users.

## Background

Tumors with predominant or partial smooth muscle differentiation make up a broad spectrum of mesenchymal neoplasms. Most of these tumors are based on spontaneous mutations of a mesenchymal stem cell. A rare and virus-associated entity are the post-transplant smooth muscle tumors (PTSMT) [1, 2]. These tumors are typically positive for the Epstein–Barr virus (EBV) and can manifest themselves at any time and in any organ after transplantation [1]. Similar neoplasms can also occur in any other immunosuppressive condition, in particular after infection with human immunodeficiency virus (HIV; HIV-SMT) or congenital immune defects (CI-SMT) [1, 3, 4].

Post-transplant smooth muscle tumors are rare in regard to the total population (< 1% of transplanted patients [5]) but represent an important clinical and radiological differential diagnosis in immune-compromised patients, particularly to post-transplant lymphoproliferative diseases (PTLD). In PTSMT, the site of manifestation determines the patients’ outcome [2]. In particular, cerebral PTSMT has a poor prognosis, while histological features (mitotic rate, cellular atypia, and necrosis) have no prognostic impact [1].

Epstein–Barr virus is specialized to infect B cells and almost all adults harbor a small population of EBV+ non-neoplastic B cells, which are controlled by T cell homeostasis. It is not known how EBV enters non-B cell tissues, such as mesenchymal cells. It is thought that the PTSMT cell of origin is derived from a perivascular non-endothelial mesenchymal smooth muscle stem cell. EBV+ PTSMT have a type III-like latency with expression of EBV protein EBNA (Epstein–Barr nuclear antigen) but often lack EBV latent membrane protein 1 (LMP1) expression [6]. The tumorigenic role of EBV in smooth muscle neoplasms is not clear because particularly HIV-SMT can be EBV negative, indicating that the virus is not absolutely necessary for aberrant smooth muscle proliferation [1].

In our previous works, we found a molecular microenvironment which is not related to EBV infection, but rather to smooth muscle differentiation [5]. In addition, EBV is able to induce neoangiogenesis, but we found only minor changes on the transcriptional level, including increased levels of angiopoietin 2 (ANGPT2) [7]. Defining driver mutations in PTSMT are still not known. However, we and others found up-regulation of MYC proto-oncogene transcripts, protein expression of phosphorylated mechanistic target of rapamycin kinase (MTOR) and phosphorylated AKT serine/threonine kinase 1 (AKT1) signaling factors [2, 6].

In the present analyses we wanted to asses if and which transcript profile of mesenchymal stem cell and signaling factors differ between PTSMT and other neoplasms with smooth muscle differentiation.

## Methods

### Tumor samples

EBV+ PTSMTs comprised six tumors from five patients; one patient had a tumor in the spleen and another in a cerebral sinus. We previously reported the clinical and histopathological characteristics of four of these patients [2]. The fifth patient was a female (age: 61 years) who developed a PTSMT in the liver 5 years after kidney transplantation (tumor diameter 3.1 cm, 10% Ki67, positive for EBV in situ hybridization (EBER), smooth muscle actin, caldesmon and desmin).

**Fig. 1:**
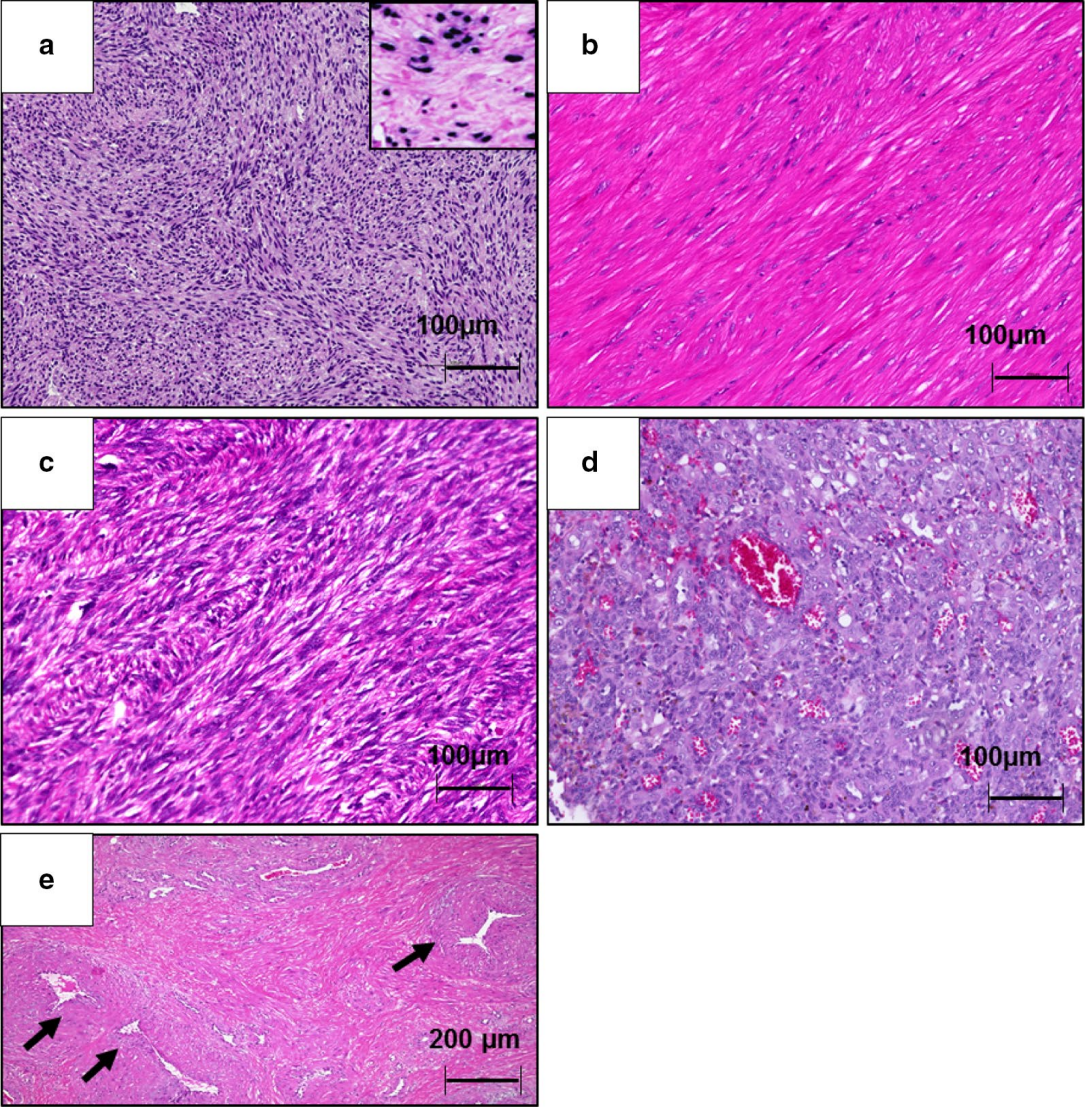
Histological appearance of the five different entities. **a** Post transplant smooth muscle tumor (haematoxylin–eosin stain (HE stain, 100×) with inserted positive EBV-in situ hybridization (EBER), **b** visceral leiomyoma (HE stain, 100×), **c** visceral leiomyosarcoma of the central venous tract (right atrium, HE stain, 100×) with prominent atypia and increased level of mitosis figures, **d** endothelial haemangioma (HE stain, 100×) with prominent vessels, **e** angioleiomyoma (HE stain, 50×), in particular venous subtype with prominently walled vessels (arrows)

For control purposes, the following tumors were analyzed (Table 1, Fig. 1): (i) smooth muscle tumors (nine visceral leiomyomas and four central venous G1–2 leiomyosarcomas) and (ii) mixed smooth muscle and vascular tumors (28 with different histological subtypes of angioleiomyomas). Additionally, we added (iii) a vascular tumor control group (five endothelial haemangiomas), as the actual origin of PTSMT is still uncertain and aberrant myogenous venous wall cells are still under discussion as the cells of origin [2, 8]. Before performing molecular analysis, the angioleiomyomas were histologically sub grouped according to the old but current standard of Morimoto [9] (13/28 capillary solid, 7/28 venous, 5/28 cavernous, 3/28 mixed capillary-cavernous). All samples of formalin fixed and paraffin-embedded (FFPE) tumors were selected from the archive of the Institutes of Pathology in Muenster (12/28 angioleiomyomas) and Hannover (all other samples).

**Table 1:** Sample set and clinical data

Entities	PTSMT	Leiomyoma (LM)	Leiomyosarcoma (LMS)	Angioleiomyoma (ALM)	Endothelial haemangioma (EHA)
Number of tumors	n = 6 (5 patients)	n = 9	n = 4	n = 28	n = 5
Age (median, range)	11.5 (6–61)	60 (29–71)	63 (45–71)	60 (23–79)	48 (32–58)
Gender	♀ 100% ♂ 0%	♀ 89% ♂ 11%	♀ 75% ♂ 25%	♀ 64% ♂ 36%	♀ 60% ♂ 40%
Tumor localisation	Kidney (n = 2) Lung (n = 1) Colon (n = 1) Spleen (n = 1) Confluens sinuum (n = 1)	Stomach (n = 3) Kidney (n = 2) Skin (n = 2) Esophagus (n = 1) Mesenterium (n = 1)	Pulmonary artery (n = 2) Right atrium (n = 1) Adrenal gland vein (n = 1)	Lower extremities (n = 13) Upper extremities (n = 2) Head–Neck (n = 3) Genital ♀ (n = 2) Skin/soft tissue (n = 8)	Lower extremities (n = 3) Head–Neck (n = 1) Mediastinal (n = 1)

### Transcript expression analysis

Samples contained > 80% tumor tissue and RNA was extracted and purified with our established mineral oil method and RNeasy Mini Kit (Qiagen, Hilden, Germany) via Maxwell^®^ system (Promega cooperation, Mannheim, Germany). RNA was quantified by Qubit^®^ 2.0 fluorometer (Life Technologies/Thermo Fisher Scientific, Waltham, MA, USA). Transcript expression analysis was performed via NanoString nCounter™ system. NanoString uses digital fluorescent reporters for gene detection and enables analysis of small amount of RNA while minimizing background signals. We used the prefabricated stem cell panel (includes 193 target genes and six endogenous control genes) as described in the manufacturer’s protocol (NanoString Technologies, Seattle, WA, USA). Endogenous control genes were glucuronidase beta (GUSB), glyceraldehyde-3-phosphate dehydrogenase (GAPDH), clathrin heavy chain (CLTC), hypoxanthine phosphoribosyltransferase 1 (HPRT1), phosphoglycerate kinase 1 (PGK1) and tubulin beta class I (TUBB).

### Immunohistochemistry

Deparaffinized and rehydrated FFPE tissue sections (1–2 μm) were processed in an automated staining system (Benchmark ULTRA, Ventana Medical Systems, Inc., Tucson, AZ, USA). A monoclonal anti-beta-catenin antibody was used.

### Data analysis

Raw data of transcript target gene expression were analyzed by nSolver™ software (NanoString Technologies) and set in relation to the average of endogenous control genes (geometric mean of controls as level of relative gene expression: reference gene index). Further statistical analysis for comparison of different tumor groups was performed with Prism 5.0 (Graph Pad Software, San Diego, CA, USA) by using the non-parametric Mann–Whitney test for two group comparison and the Krustal–Wallis test and post hoc Dunn-test for multiple comparisons. P values < 0.05 were considered as statistically significant. Heatmaps were generated using R.

## Results

### Increased WNT6/WNT10A levels in PTSMT

**Fig. 2:**
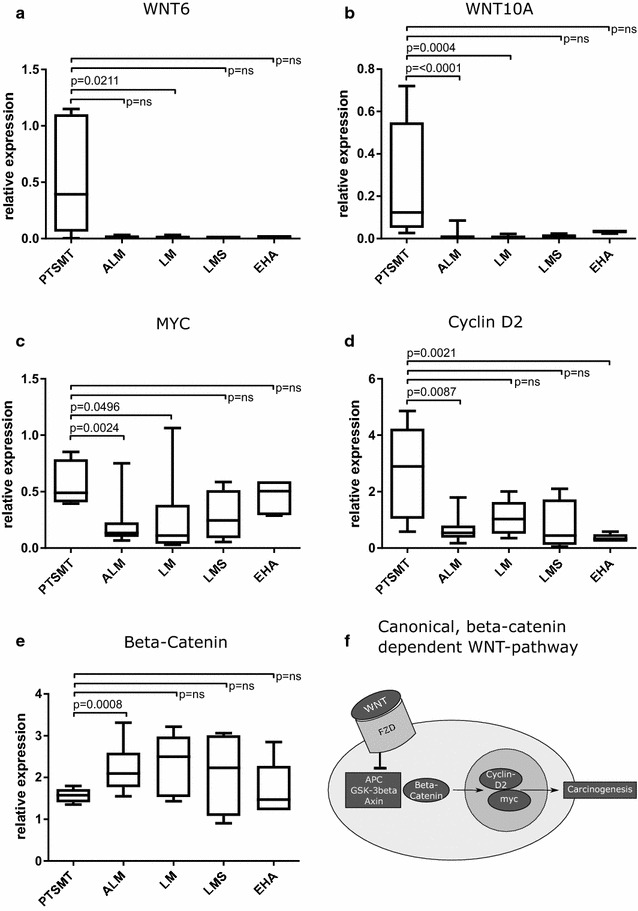
**a**–**e** Significant upregulated gene members of the WNT signaling pathway in PTSMT. Note that non-significant (p = ns) differences between PTSMT and other tumors regarding WNT6 and WNT10A expression are related to non-detactable transcripts in several ALM, LMS and EHA (**a**, **b**). **f** Beta-catenin is bound and inactivated in a complex formation containing adenomatous polyposis coli gene (APC), glycogen synthase kinase 3 beta (GSK-3beta) and axin-1 (Axin). In the classical, beta-catenin-dependent WNT signaling pathway, members of WNT family bind to the frizzled class receptors (FZD) whereas beta-catenin is set free and initiates carcinogenesis via activation of MYC and Cyclin D2

Transcript analysis showed significant differential expression of several genes among the different types of smooth muscle tumors (Table 2, Additional file 1: Figure S1). Several factors were increased in only one type of tumor. PTSMT showed increased Wnt family member 6 (WNT6) and WNT10A levels while all other types of tumors showed no different expression or low levels of these two WNT factors (Fig. 2, Table 2). WNT6 and WNT10A are encoded as a gene cluster on chromosomal segment 2q35. In individual PTSMT cases, both WNT factors were co-expressed at similar levels.

**Table 2:** Significant differentially regulated genes in PTSMTs and leiomyosarcomas

Regulation	Gene	Median PTSMT	Median ALM	Median LM	Median LMS	Median EHA
Upregulated in PTSMT	WNT6	0.39301	0.00961	0.00439	0.01074	0.01285
	WNT10A	0.12282	0.00545	0.00482	0.00670	0.03415
	MYC	0.48877	0.13366	0.11219	0.24502	0.50480
	Cyclin D2	2.89498	0.54088	1.02805	0.44413	0.32572
Downregulated in PTSMT	WNT9B	0.00192	0.00416	0.00193	0.00951	0.01138
	GAS1	0.02407	0.05434	0.19764	0.28556	0.30450
	PRKD1	0.07241	0.21601	0.22282	0.15769	0.12693
	FGFR1	0.36842	0.80905	1.72532	0.93143	0.48040
	Beta-catenin	1.57460	2.09349	2.49687	2.23053	1.47197
Upregulated in leiomyosarcomas compared to PTSMT	WNT9B	0.00192	0.00416	0.00193	0.00951	0.01138
	GAS1	0.02407	0.05434	0.19764	0.28556	0.30450
	DHH	0.00297	0.01077	0.00831	0.00259	0.04553
	LFNG	0.23836	0.77146	0.22185	0.76354	2.24482
	MFNG	0.05390	0.07672	0.05256	0.05478	0.50480
	PRKACA	0.85552	1.53877	1.51137	1.44838	1.70591
	IGF	0.04295	0.12047	0.11971	0.16677	0.67152

In addition to WNT6/WNT10A, cell cycle G1/S transition regulator Cyclin D2 (CCND2) was increased. As could be expected from our previous results on uterine leiomyomas and PTSMT [2], MYC proto-oncogene, bHLH transcription factor (MYC) transcripts were significantly higher in PTSMT than in non-uterine visceral leiomyomas (Fig. 2). These three factors are not encoded on 2q35 or any other chromosome 2 segment, but on chromosome 8 (MYC: 8q24) and chromosome 12 (CCND2: 12p13).

WNT9B (17q21.32), S phase entry blocker growth arrest specific 1 (GAS1) and serine/threonine protein kinase D1 (PRKD1) were expressed at low levels in PTSMT while the other analyzed tumor types showed higher levels, in particular, leiomyosarcomas. Similar to our previous results [7], fibroblast growth factor receptor 1 (FGFR1) levels are lower in PTSMT as compared to leiomyomas.

WNT signaling-related transcript levels of catenin beta 1 (CTNNB1) were not increased in PTSMT (Fig. 2, Table 2). Beta-catenin protein expression was evaluated by immunohistochemistry in PTSMT. All of them showed no nuclear beta-catenin protein localisation. Compared to the other tumor types, several other canonical and non-canonical WNT signaling pathway factors were not significantly deregulated in PTSMT, in particular, frizzled class receptors (FZD), WNT1-8, WNT9A, WNT10B, WNT11 and WNT16, beta-catenin-related transcription factor 7 (TCF7), WNT signaling pathway regulator adenomatous-polyposis-coli gen (APC), glycogen synthase kinase 3 beta (GSK-3beta), Cyclin D1 (CCND1) as well as the two small GTPase ROH family members ras homolog family member A (ROHA) and cell division cycle 42 (CDC42). These findings indicate that other canonical and non-canonical WNT pathways are not activated and affirm our theory. Therefore, the downstream effectors of the elevated WNT6/WNT10A levels remain unclear.

### Different gene expression profiles in PTSMT and leiomyosarcomas

There were significant differences between visceral leiomyomas, angioleiomyomas and haemangiomas (Additional file 1: Figure S1).

In comparison to all other tumors, leiomyosarcomas showed several increased gene expression levels (Table 2). The following expression levels were significantly higher in leiomyosarcomas than in PTSMT (Table 2): WNT9B, GAS1, signaling factor desert hedgehog (DHH), the two Notch signaling pathway factors LFNG O-fucosylpeptide 3-beta-N-acetylglucosaminyltransferase provided (LFNG) and MFNG O-fucosylpeptide 3-beta-N-acetylglucosaminyltransferase (MFNG), protein kinase cAMP-activated catalytic subunit alpha (PRKACA) and insulin-like growth factor 1 (IGF1). Roland et al. [10] showed that leiomyosarcomas also express the IGF receptor which indicates paracrine activation.

### Histopathological subgroups of angioleiomyomas show no molecularpathological correlation

There are three distinct histopathological subtypes of angioleiomyomas which were originally described on the basis of histopathological features by Morimoto in 1973 [9]. Regarding the genes under investigation, manly these subgroups showed the same molecularpathological characteristics. Only six genes show significant differences between the angioleiomyoma types (ISL1, NCSTN, TCF7, WNT10A, WNT11, WNT7A) but their relative expression levels were very low, which indicates no biological relevance (< 0.4 compared to standardized reference gene index).

## Discussion

Post-transplant smooth muscle tumor share morphological similarities with leiomyomas and low-grade leiomyosarcomas and only show rarely high-grade atypia. One of the main differences is the EBV association. Our previous molecular analysis showed no clear relationship between EBV infection of tumor cells and (de)regulation of gene expression. One reason could be that PTSMT usually do not express LMP1, a viral protein which is associated with manipulation of the host cell cycle and cytokine expression. In one of the very first analyses which addressed the question of specific signaling pathway deregulation in PTSMT, Ong et al. [6] demonstrated that these tumors express phosphorylated MTOR and phosphorylated AKT1 proteins and that Ras association domain family member 1 (RASSF1) was hypermethylated. Activated MTOR/AKT1 signaling, RASSF1 hypermethylation and MYC expression can also be found in a subset of leiomyosarcomas [6, 11, 12]. We could confirm our previous result that PTSMT have higher expression levels of MYC than uterine and non-uterine leiomyomas. In our current analysis we found that this difference regarding MYC was not significant in comparison to leiomyosarcomas. Remarkably, for the very first time, we could show that PTSMT are characterized by WNT6/WNT10A regulation while all other tumor entities under investigation showed almost no expression of these two factors. There are no reports on a particular WNT6 or WNT10A expression in EBV infected cells [13, 14]. Therefore, it is unlikely that EBV is the main cause of this WNT6/WNT10A expression. The only other known tumor entity characterized by WNT6/WNT10A overexpression are colorectal carcinomas and it is assumed that their mode of proliferation is linked to differential beta-catenin expression—as opposed to PTSMTs [15].

Both genes are encoded as a cluster on segment 2q35. Co-expression of these two factors indicates a co-regulation of the respective promotors. The fact that many canonical WNT signaling factors were not increased and that we found no nuclear beta-catenin indicates activation of the non-canonical WNT pathway. Similar to PTSMT, leiomyosarcomas usually show no aberrant nuclear beta-catenin expression [10, 16]. Non-canonical WNT signaling is often characterized by alternative signaling without cytoplasmic stabilization of soluble beta-catenin [17–19]. These include the WNT/calcium pathway and the planar cell polarity (PCP). In those alternative pathways, other WNT factors than WNT6 and WNT10A are usually involved [17–19]. In contrast to PTSMT, it has been shown in renal cell carcinoma that WNT10A is involved in canonical WNT/beta-catenin signaling [20]. In the murine kidney, Wnt6 is also related to canonical Wnt/beta-catenin signaling [21]. In mesenchymal cells expression of WNT6, WNT10A and WNT10B can be associated with osteoblastogenesis and inhibition of adipogenesis but not with smooth muscle differentiation [22]. WNT6 is involved in differentiation of non-smooth muscle myogenic cells, e.g. in the heart, and proliferation of stromal cells in the placenta [23–26]. There are reports which imply a special relationship between WNT/beta catenin signaling and CCND2 in glioma cells [27] but not in smooth muscle cells. At least in cardiomyocytes, there are evidences of a MYC-dependent activation of CCND2 [28]. In insulin sensitive pancreatic beta cells, activated MTOR leads to CCND2-associated proliferation [29, 30]. Therefore, these signaling factors could also be linked in PTSMT. Based on the finding that MTOR signaling is activated, Sirolimus has been used for targeted therapy in PTSMT [31].

## Conclusions

In summary, we are the first to report that PTSMT cells may harbor a unique variant of a non-canonical WNT pathway, which is based on WNT6/WNT10A, and which may induce cell proliferation and differentiation via MTOR/AKT1, MYC and CCND2.

## Additional file

**Additional file 1: Figure S1.** Heatmap of all investigated stem cell genes shows differences between the five entities. PTSMT: post-transplant smooth muscle tumors, ALM: angioleiomyomas and their histomorphological subtypes, LM: leiomyomas, EHA: endothelial haemangiomas and LMS: leiomyosarcomas of the central venous tract. Colors encode significance level of pairwise group comparison. The abbreviations of the five entities stand for the corresponding significantly different regulated group. ns: p > 0.05, *: p ≤ 0.05, **: ≤0.01, ***: ≤0.001.

## Abbreviations

AKT1: serin/threonin kinase 1
ANGPT2: angiopoietin 2
APS: adenomatous-polyposis-coli gen
CCND1: Cyclin-D1
CCND2: Cyclin-D2
CDC42: cell division cycle 42
CI-SMT: congenital immune defects-smooth muscle tumors
CLTC: clathrin heavy chain
CTNNB1: beta-catenin 1
DHH: desert hedgehog
EBER: Epstein–Barr virus
EBNA: Epstein–Barr nuclear antigen
EBV: Epstein–Barr virus-encoded small RNA
FFPE: formalin fixed and paraffin-embedded
FGFR1: fibroblast growth factor receptor 1
FZD: frizzled class receptor
GAPDH: glyceraldehyde-3-phosphate dehydrogenase
GAS1: S phase entry blocker growth arrest specific 1
GSK-3beta: glycogen synthase kinase 3 beta
GUSB: glucuronidase beta
HE: hematoxylin and eosin
HIV: human immunodeficiency virus
HIV-SMT: human immunodeficiency virus smooth muscle tumors
HPRT1: hypoxanthine phosphoribosyltransferase 1
IGF: insulin like growth factor 1
ISL1: insulin gene enhancer protein
LFNG: LFNG *O*-fucosylpeptide 3-beta-*N*-acetylglucosaminyltransferase provided
LMP1: latent membrane protein 1
MFNG: MFNG *O*-fucosylpeptide 3-beta-*N*-acetylglucosaminyltransferase
MTOR: rapamycin kinase
NCSTN: nicastrin
PCP: planar cell polarity
PGK1: phosphoglycerate kinase 1
PRKACA: protein kinase cAMP-activated catalytic subunit alpha
PRKD1: serine/threonine protein kinase D1
PTLD: post-transplant lymphoproliferative disease
PTSMT: post-transplant smooth muscle tumor
RASSF1: Ras associated domain family member 1
ROHA: GTPase ROH family members ras homolog family member A
TCF7: beta-catenin-related transcription factor 7
TUBB: tubulin beta class 1
WNT6: WNT family gene member 6
WNT10A: WNT family gene member 10A
