# Supplementary figures and images for: Non-canonical WNT6/WNT10A signal factor expression in EBV+ post-transplant smooth muscle tumors
Source: Clin Sarcoma Res. 2018 Jun 4;8:10. doi: 10.1186/s13569-018-0096-8 (PMC5985559; doi:10.1186/s13569-018-0096-8)

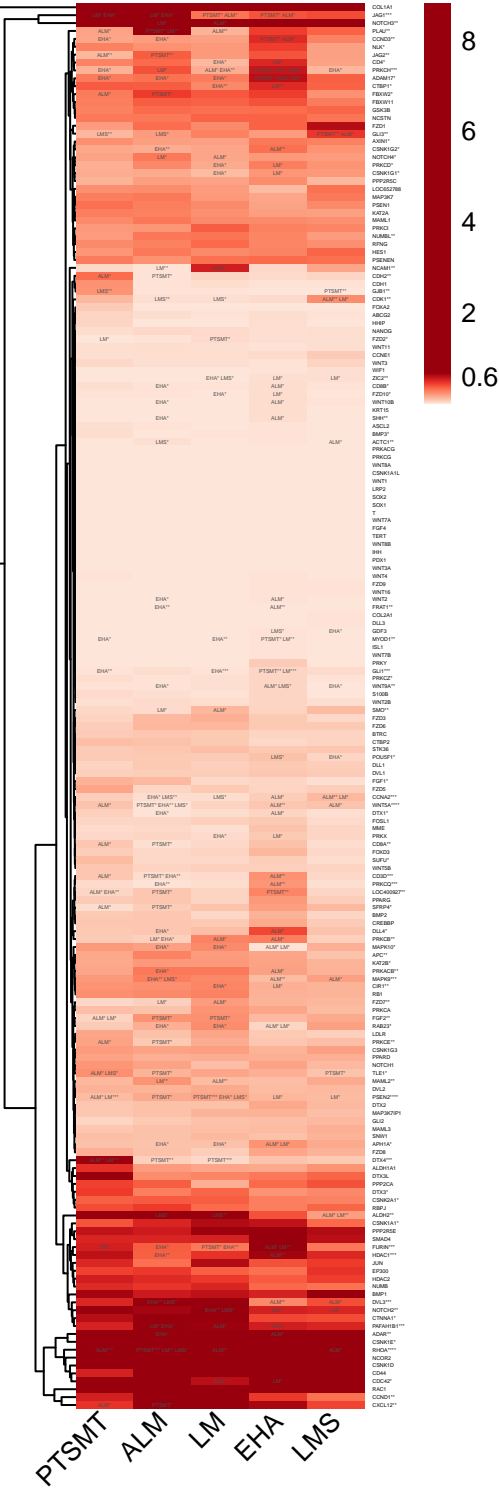

Supplement: Supplementary file 1 — Additional file 1: Figure S1. Heatmap of all investigated stem cell genes shows differences between the five entities. PTSMT: post-transplant smooth muscle tumors, ALM: angioleiomyomas and their histomorphological subtypes, LM: leiomyomas, EHA: endothelial haemangiomas and LMS: leiomyosarcomas of the central venous tract. Colors encode significance level of pairwise group comparison. The abbreviations of the five entities stand for the corresponding significantly different regulated group. ns: p > 0.05, *: p ≤ 0.05, **: ≤0.01, ***: ≤0.001. [file 13569_2018_96_MOESM1_ESM.pdf]
